# Influence of the blood bacterial load on the meningeal inflammatory response in *Streptococcus pneumoniae *meningitis

**DOI:** 10.1186/1471-2334-6-78

**Published:** 2006-04-27

**Authors:** Christian ∅stergaard, Terence O'Reilly, Christian Brandt, Niels Frimodt-Møller, Jens D Lundgren

**Affiliations:** 1 Division of Microbiology, National Center for Antimicrobials and Infection Control, Statens Serum Institut, Copenhagen, Denmark; 2 Novartis Institute for Biochemical Research, Basel, Switzerland; 3 CHIP, Hvidovre University Hospital, Hvidovre, Denmark

## Abstract

**Background:**

Despite bacteraemia is present in the majority of patients with pneumococcal, little is known about the influence of the systemic infection on the meningeal inflammatory response.

**Methods:**

To explore the role of systemic infection on the meningeal inflammation, experimental meningitis was induced by intracisternal injection of ~1 × 10^6 ^CFU *Streptococcus pneumoniae*, type 3, and the 26 rabbits were either provided with ~1 × 10^6 ^CFU *S. pneumoniae *intravenously at 0 hour ("bacteraemic" rabbits, n = 9), immunized with paraformaldehyde-killed *S. pneumoniae *for 5 weeks prior to the experiment ("immunized" rabbits", n = 8), or not treated further ("control" rabbits, n = 9). WBC and bacterial concentrations were determined in CSF and blood every second hour during a 16 hours study period together with CSF IL-8 and protein levels. We also studied CSF and blood WBC levels in 153 pneumococcal meningitis patients with and without presence of bacteraemia.

**Results:**

As designed, blood bacterial concentrations were significantly different among three experimental groups during the 16 hours study period (Kruskal Wallis test, *P *< 0.05), whereas no differences in CSF bacterial levels were observed (*P *> 0.05). Blood WBC decreased in bacteraemic rabbits between ~10–16 hours after the bacterial inoculation in contrast to an increase for both the immunized rabbits and controls (*P *< 0.05). The CSF pleocytosis was attenuated in bacteraemic rabbits as compared to the two other groups between 12–16 hours from time of infection (*P *< 0.017), despite accelerated CSF IL-8 levels in bacteraemic rabbits.

In patients with pneumococcal meningitis, no significant difference in CSF WBC was observed between patients with or without bacteraemia at admission (n = 103, 1740 cells/μL (123–4032) vs. n = 50, 1961 cells/μL (673–5182), respectively, *P *= 0.18), but there was a significant correlation between CSF and blood WBC (n = 127, Spearman rho = 0.234, *P *= 0.008).

**Conclusion:**

Our results suggest that a decrease in peripheral WBC induced by enhanced bacteraemia in pneumococcal meningitis results in an attenuated CSF pleocytosis.

## Background

*Streptococcus pneumoniae *meningitis is characterized by an accumulation of leukocytes within the subarachnoidal space. It has been suggested that host inflammatory reactions to the invading pathogen are largely responsible for the poor outcome of pneumococcal meningitis, because pathophysiological alterations leading to death continue to evolve after the bacterial eradication by antibiotic therapy [1,2]. Therefore, the mechanisms behind the regulation of the pleocytosis have been studied carefully during the last three decades, and adjunctive therapy has predominantly been designed to decrease the harmful effect of the meningeal inflammatory response (e.g. dexamethasone in clinical trials [3] and various anti-inflammatory strategies in preclinical testing [1,2]). Recent studies may, however, reveal a less important role of the meningeal inflammatory response and a more significant role of the systemic infection/inflammation on the mortality of pneumococcal meningitis, i.e: 1) patient with a low number of CSF WBC were at an increased risk of dying from pneumococcal meningitis [4]. 2) Blocking of leukocyte entry into the CSF increased the mortality in experimental pneumococcal meningitis most likely due to an augmented bacteraemia [5]. 3) Bacteraemia was present in 2 of every three patient with pneumococcal meningitis, and a large number of these patients had septic shock with systemic complications accounting for approximately half of the death from the disease [4]. 4) The beneficial effect of anti-inflammatory therapy with dexamethasone was predominantly observed on systemic complications than on neurological complications [6].

Cytokine expression is widely upregulated in the brain after intravenously LPS injection [7], but no pleocytosis is observed, and CSF cytokine levels are either not detectable or significant lower than the corresponding blood concentrations [8]. Conversely, cytokines are locally released in the CSF during meningitis [8,9] with undetectable or insignificant corresponding blood levels, which had led to the current opinion that the meningeal inflammatory response is not influenced by the events taking place within the systemic compartment. On the other hand, no studies have to our knowledge addressed the influence of an altered blood bacterial load on the meningeal inflammatory response during meningitis. Moreover, alterations in the systemic inflammatory response (e.g. leukopenia induced by cyclophosphamide [10,11], depletion of systemic monocytes [12], increase in number of neutrophils by G-CSF pretreatment [9]) showed an influence on the meningeal inflammatory response during experimental pneumococcal meningitis. Thus, several aspects of how the systemic infection/inflammation influences the meningeal inflammatory response during pneumococcal meningitis still remain to be defined.

The aim of the present study was hence to examine to which extend the presence of bacteraemia affects the CSF pleocytosis in patients with pneumococcal meningitis and during the early course of experimental pneumococcal meningitis. To enhance the level and onset of bacteraemia during the course of experimental meningits, pneumococci were concomitantly injected intravenously, and to remove the bacteraemia that accompanies the meningitis, we used active immunization of rabbits.

## Methods

### Experimental pneumococcal meningitis

For the meningitis experiments, rabbits were infected intracisternally with ~1 × 10^6 ^CFU serotype 3 *S. pneumoniae*, essential as previous described [9]. CSF and blood samples were obtained every second hour during a 16-hours study period and were analyzed for bacterial concentrations by quantitative cultures (lower detection level: 50 CFU/ml) and WBC on an automatic cell counter (Swelab, Åstad, Sweden). After centrifugation, CSF supernatants were analyzed for IL-8 using a rabbit specific ELISA and for protein content using the Lowry method [9].

### Study design

In addition to the induced meningitis, rabbits were divided into three groups: 1) "Bacteraemic" comprised of 9 rabbits that were given ~1 × 10^6 ^CFU serotype 3 *S. pneumoniae *intravenously at the time of the intracisternal inoculation. 2) "Immunized" comprised of 8 immunized rabbits. To obtain a significant humoral response against pneumococci, the rabbits were immunized by intravenously injection of paraformaldehyde-killed serotype 3 *S. pneumoniae *(1 ml of a 3–6 × 10^9 ^CFU/ml bacterial suspension (Statens Serum Institut, vaccine lot. #E31), three times per week for ~5 weeks). The serotiter was determined after the immunization has ended and before the start of the meningitis experiment, using the Neufeld capsule reaction [13]. In brief, a two-fold dilution in isotonic NaCl of serum obtained from immunized rabbits was mixed together with 6 × 10^9 ^CFU/ml serotype 3 *S. pneumoniae *(1:1). The serotiter was determined as the reciprocal of the highest dilution with a visible quelling reaction and was found to be 4 (n = 1), 8 (n = 6) and 16 (n = 1). 3) Controls comprised of 9 infected control rabbits without additional intervention.

### Patients with pneumococcal meningitis

153 patients with pneumococcal meningitis, who had available CSF WBC counts from a diagnostic lumbar puncture taken at admission, were included. The 153 patients were part of 187 consecutive patients from a Danish nationwide study, and patients characteristics has been presented in detail previously [4]. All 153 patients had available blood culture data, whereas 127 out of 153 patients had available blood WBC data. Meningitis was defined as a CSF culture with *S. pneumoniae *or CSF pleocytosis (>10 leukocytes/mL) in conjunction with a blood culture of *S. pneumoniae*.

### Ethics

All human protocols were approved by the local scientific ethic committee and the Danish Data Protection Agency (#2002-41-2278). The Danish Animal Experiment Inspectorate (Dyreforsøgstilsynet) approved all experimental protocols.

### Statistically analysis

All results are given as medians (25/75 percentiles). Comparison between the 3 experimental groups was performed by Kruskal-Wallis Test, and when significant it was followed by Mann-Whitney test with Bonferroni's correction for multiple comparisons. Multivariate ANOVA (MANOVA) with repeated measures was used on selected data to further explore generated relations. Correlation between parameters was performed by non-parametic Spearman rank test. *P *< 0.05 was considered significant, except when Bonferronis correction was used (*P *< 0.017). GraphPad Prism, version 4.01, GraphPad Software Inc., San Diego, CA, USA was used for all statistical analysis except MANOVA (SAS/JMP Software, version 5.1, SAS institute Inc, Cary, NC, USA).

## Results

### Experimental pneumococcal meningitis

**Figure 1:**
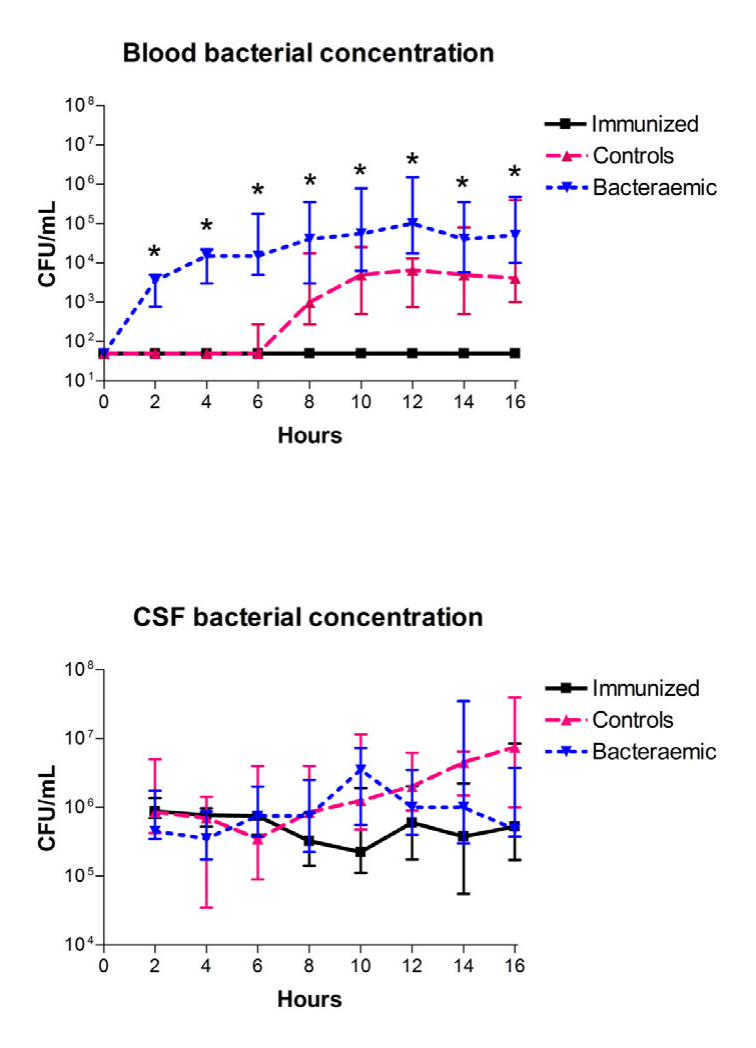
**CSF and blood bacterial concentration during experimental pneumococcal meningitis**. *Significant difference between groups (Kruskal Wallis test, *P *< 0.05). There was a significant higher blood bacterial concentration in bacteraemic rabbits (n = 9) than in controls (n = 9) at 2–6 and 12 hours and than in immunized rabbits (n = 8) at 2–16 hours, respectively, and higher in controls than in immunized rabbits at 10–16 hours (Mann Whitney test, *P *< 0.017). All data are shown as medians (25–75 percentiles).

As per the study design, blood bacterial concentrations were significantly different between the three groups at 2–16 hours after the bacterial inoculation (Figure 1, top, Kruskal Wallis test, *P *< 0.05). All bacteraemic and control rabbits developed secondary bacteraemia, whereas immunized rabbits never develop bacteraemia during the 16-hour study period (Figure 1, top). The bacteraemic group developed detectable bacteraemia earlier (at ~2 hours) than control rabbits, where bacteraemia was first seen after 6 hours, thus having consistently higher blood bacterial concentrations than control rabbits during the 16 hours study period. Subsequent MANOVA analysis confirmed these results (data not shown).

### CSF bacterial concentrations

No statistically significant difference in CSF bacterial concentrations was found between the 3 groups during the 16-hour study period (Figure 1, bottom, Kruskal Wallis test, *P *> 0.05). Subsequent MANOVA analysis confirmed these results and overall, there was no significant influence of group (MANOVA, *P *= 0.418), but there was an effect of time and interaction between groups and time (both MANOVA. *P *< 0.001). Based upon comparisons to 2 h post CSF infection, bacteraemic rabbits had initially the highest growth rate (~1 Log_10 _CFU/mL between 2–10 hours), whereas between 10–16 hours a net decrease in CSF bacterial concentrations were observed. In contrast, immunized rabbits had apparently no significant bacterial growth within the CSF, whereas controls had a steady increase in CSF bacterial concentrations of ~1 Log_10 _CFU/mL during the 16-hour study period. This difference between groups reached significance by hour 14 (MANOVA, *P *< 0.003), and at 16 hours the immunized group had a significantly lower CSF bacterial concentrations than the control group (MANOVA, *P *= 0.008).

### CSF and blood WBC concentrations

**Figure 2:**
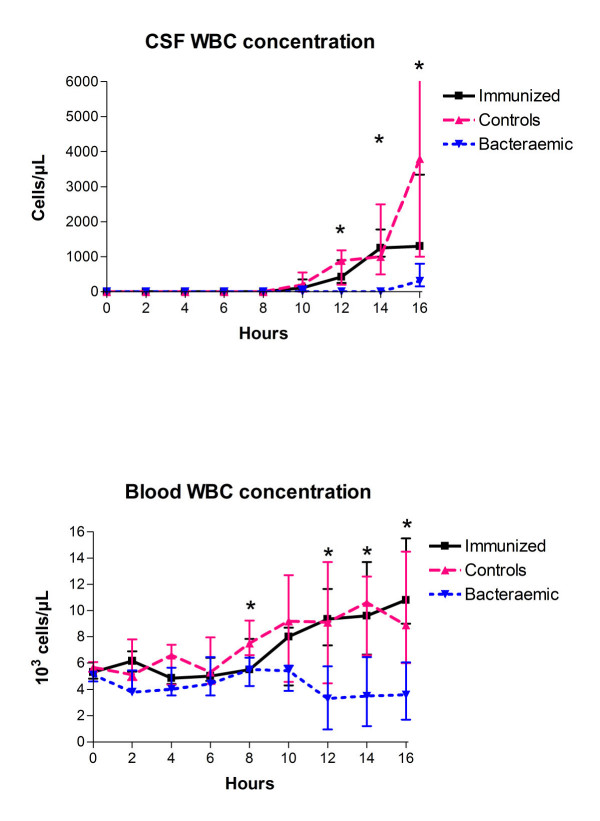
**CSF and blood WBC concentration during experimental pneumococcal meningitis**. *Significant difference between groups (Kruskal Wallis test, *P *< 0.05). Bacteraemic rabbits (n = 9) had significantly lower CSF WBC counts as compared to controls (n = 9) and to immunized rabbits (n = 8) between 12–16 hours (Mann Whitney test, *P *< 0.017). Bacteraemic rabbits had significantly lower blood WBC levels as compared to controls at 8 hours and between 12–16 hours and to immunized rabbits between 12–16 hours, respectively (Mann Whitney test,*P *< 0.017). All data are shown as medians (25–75 percentiles).

CSF WBC counts were significantly different between the 3 groups from start of the pleocytosis at ~10–12 hours until the end of the 16-hours study period (Figure 2, top, Kruskal Wallis test, *P *< 0.05). Bacteraemic rabbits had significantly lower CSF WBC counts as compared to controls and to immunized rabbits (Mann-Whitney test, *P *< 0.017), whereas no statistically significant difference was observed between controls and immunized rabbits (Mann Whitney test, *P *> 0.017). Similarly, the blood WBC counts varied significantly between the three groups from 8 hours and onwards with significant difference between the three groups at 8 hours and between 12–16 hours (Figure 2, bottom, Kruskal Wallis test, *P *< 0.05). Controls had an elevation in blood WBC counts from ~8 hours after the bacterial inoculation, immunized rabbits developed leukocytosis slightly later than control rabbits, however with no statistical differences between the two groups (Mann Whitney test, *P *> 0.017). Conversely, bacteraemic rabbits developed a consistent decrease in blood WBC counts from 10 hours with significantly lower WBC levels as compared to controls at 8 hours and between 12–16 hours and to immunized rabbits between 12–16 hours, respectively (Mann Whitney test, *P *< 0.017). Subsequent MANOVA analysis confirmed these results (data not shown).

Not surprisingly, there was a significant correlation between CSF and blood WBC over time or when all three groups were included in the analysis due to alterations in these parameters over time or between groups (data not shown). However, there was a positive correlation between CSF and blood WBC (rho>0.38), reaching statistical significance at 14 hours (p = 0.001), when analysing the two groups that were not significantly different (controls and immunized rabbits), regarding CSF and blood WBC levels.

### CSF IL-8 and protein concentrations

**Figure 3:**
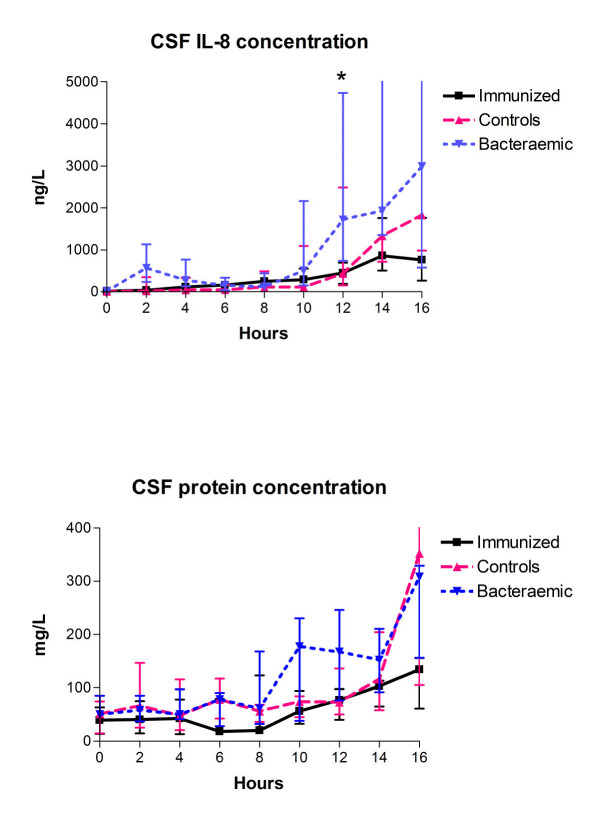
**CSF IL-8 and protein concentration during experimental pneumococcal meningitis**. All data are shown as medians (25–75 percentiles). *Significant difference between groups (Kruskal Wallis test, *P *< 0.05).

CSF IL-8 levels were higher in bacteraemic rabbits as compared to immunized rabbits and control rabbits (Figure 3, top) from ~12–16 hours after the bacterial inoculation, however with only statistical significance between bacteraemic and immunized rabbits at 12 hours (1718 ng/L (735–4731) vs. 450 ng/L (188–693), respectively, Mann Whitney test, *P *= 0.011). Compared to hour 0 all groups had an increase in CSF IL-8 levels during the study period, and at 16 hours there was a significant difference between groups (MANOVA, *P *< 0.023). No significant difference in CSF protein levels was observed between the three groups during the 16-hour study period (Figure 3 bottom, Kruskal Wallis test, *P *> 0.05). Subsequent MANOVA analysis confirmed these results (data not shown).

### Patients with pneumococcal meningitis

**Figure 4:**
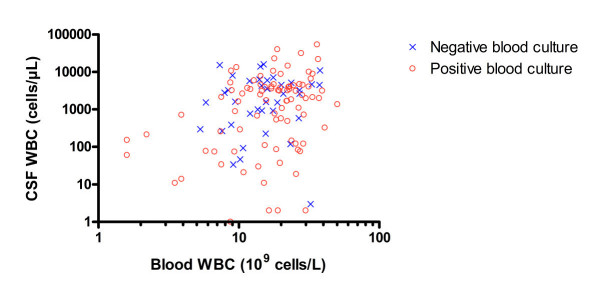
**Correspondent CSF and blood WBC concentrations in patients with pneumococcal meningitis**. Correlation between CSF and blood WBC: all patients (n = 127, rho = 0.234, *P *= 0.008), patients with a positive blood culture (n = 89, rho = 0.272, *P *= 0.01) and patients with a negative blood (n = 38, rho = 0.159, *P *= 0.34). No significant difference in CSF WBC between patients with a positive or a negative blood culture (*P *> 0.05).

Positive CSF cultures were detected in 146 out of 153 patients (95%), whereas a positive blood culture was present in 103 out of 153 patients with pneumococcal meningitis (67%). There was no significant difference in CSF WBC between patients with a positive or a negative blood culture (1740 cells/μL (123–4032) vs. 1961 cells/μL (673–5182), respectively, Mann Whitney test, *P *= 0.18) in contrast to a significant correlation between CSF WBC and blood WBC (Figure 4, n = 127, Spearman test, rho = 0.234, *P *= 0.008). No significant association was found between other CSF routine parameters (protein – and glucose levels) and blood WBC counts/presence of bacteraemia (data not shown).

## Discussion

In the present study, inducing bacteraemia during the early part of experimental pneumococcal meningitis was associated with attenuation of the development of CSF pleocytosis despite accentuated concentrations of the chemokine IL-8 in the CSF. The attenuated CSF pleocytosis associated with the early onset bacteraemia was likely due to a decrease in blood WBC induced by the bacteraemia as opposed to directly caused by the bacteraemia *per se*, since the avoidance of bacteraemia by active immunization against pneumococci did neither affect the CSF pleocytosis nor blood leukocytosis as compared to controls. Further supporting the explanation that the decrease in blood WBC, induced by the early onset bacteraemia, was responsible for the attenuated CSF pleocytosis observed experimentally, we found a significant correlation between blood WBC and CSF WBC counts in patients with pneumococcal meningitis, which was in contrast to no association between presence of bacteraemia and number of CSF WBC.

Several studies have previously documented an altered leukocyte function during endotoxemia (e.g. decreased in chemotactic activity of neutrophils toward various chemotactic signals [14] including IL-8 [15], shedding of neutrophil receptors such as IL-8-receptor [16] and L-selectin [17]), resulting in a decreased migration of leukocytes to extravascular infection foci (e.g. lung [18], peritoneum [19], skin [20]). Additionally, we have shown an attenuated pleocytosis during endotoxemia in LPS-induced meningitis [O'Reilly T, ∅stergaard C, Vaxelaire J, and Zak O: *Systemic inflammation alters the inflammatory response in experimental LPS-induced meningitis *(manuscript submitted for publication)], whereas only sparse information has been generated about the influence of pneumococcal bacteraemia on the recruitment of leukocytes to local infections sites. In contrast to our results, Sato et al. [21] found no significant difference in number of alveolar neutrophils during pneumococcal pneumonia between bacteraemic rabbits and non-bacteraemic rabbits, despite a significant decrease in peripheral neutrophils in bacteraemic rabbits, which was a result of an increased sequestration of neutrophils in the lung [22]. Not surprisingly caused an elimination of peripheral leukocytes by cyclophosphamide an attenuated pleocytosis in experimental pneumococcal meningitis [10,11], but also an altered neutrophil function with pneumococcal bacteraemia could be of importance, since a decreased chemotactic ability of neutrophils with G-CSF pretreatment [9] and a blocking of adhesion molecules [23] has been associated with an attenuated pleocytosis.

In the present study, CSF IL-8 levels were higher in bacteraemic animals, whereas no alteration in CSF IL-8 levels was found in immunized rabbits as compared to controls. Also, an attenuation of leukocyte entry into the CSF by the selectin-blocker, Fucoidin resulted in enhanced CSF IL-8 levels [23]. IL-8 is a chemokine locally produced during meningitis and has an essential role in the recruitment of neutrophils into the CSF [24,25]. Our results suggest that a novel feedback mechanism involving blood derived leukocytes that control the CSF production of IL-8 as a local chemotactic signal, which is accordance with findings from a rat pneumonia model, where MIP-2, the IL-8 analog in rats, was higher in alveolar fluid during cyclophosphamide-induced leukopenia [26]. However, a lower degree of IL-8 binding to the IL-8 receptor on CSF neutrophils could also explain the finding of higher IL-8 levels in CSF supernatants from rabbits with an attenuated pleocytosis.

CSF bacterial concentrations were not affected by alteration in the blood bacterial load confirming previous findings from the rabbit model that an enhancement of the humoral immune response did not affect the CSF bacterial load during the early phase of pneumococcal meningitis [27]. Moreover, the attenuated CSF pleocytosis in bacteraemic rabbits did not influence CSF bacterial concentrations confirming that the CNS is a compartment distinct from the systemic circulation regarding host defense mechanisms against invading bacteria [9,11,23,24]. No significant difference in CSF protein levels was observed between the three groups, indicating that the blood/brain barrier permeability was not influenced by the degree of bacteraemia, but conflicting with previous findings showing that an attenuated pleocytosis was associated with a decreased blood-brain barrier break down [23,28].

## Conclusion

We found a significant correlation between CSF WBC and blood WBC in patients with pneumococcal meningitis and that CSF pleocytosis was attenuated with a decrease in peripheral WBC as a consequence of an earlier onset bacteraemia in experimental pneumococcal meningitis.

## Competing interests

The author(s) declare that they have no competing interests.

## Authors' contributions

C∅ provided the scientific idea of the study, designed the study, and drafted the manuscript. TOR, CB, NFM, and JDL took part in preparation of the manuscript. All authors approved the final manuscript.

## Pre-publication history

The pre-publication history for this paper can be accessed here:


